# Seed Endophytic Bacteria of Pearl Millet (*Pennisetum glaucum* L.) Promote Seedling Development and Defend Against a Fungal Phytopathogen

**DOI:** 10.3389/fmicb.2021.774293

**Published:** 2021-12-09

**Authors:** Kanchan Kumar, Anand Verma, Gaurav Pal, Anubha, James F. White, Satish K. Verma

**Affiliations:** ^1^Department of Botany, Institute of Science, Banaras Hindu University, Varanasi, India; ^2^Department of Plant Biology, Rutgers University, New Brunswick, NJ, United States

**Keywords:** pearl millet, seed endophytic bacteria, plant growth promotion, biocontrol, lipopeptides, live-dead staining

## Abstract

Seed endophytic bacteria (SEB) are primary symbionts that play crucial roles in plant growth and development. The present study reports the isolation of seven culturable SEB including *Kosakonia cowanii* (KAS1), *Bacillus subtilis* (KAS2), *Bacillus tequilensis* (KAS3), *Pantoea stewartii* (KAS4), *Paenibacillus dendritiformis* (KAS5), *Pseudomonas aeruginosa* (KAS6), and *Bacillus velezensis* (KAS7) in pearl millet seeds. All the isolates were characterized for their plant growth promoting activities. Most of the SEB also inhibited the growth of tested fungal phytopathogens in dual plate culture. Removal of these SEB from seeds compromised the growth and development of seedlings, however, re-inoculation with the SEB (*Kosakonia cowanii*, *Pantoea stewartii*, and *Pseudomonas aeruginosa*) restored the growth and development of seedlings significantly. Fluorescence microscopy showed inter and intracellular colonization of SEB in root parenchyma and root hair cells. Lipopeptides were extracted from all three *Bacillus* spp. which showed strong antifungal activity against tested fungal pathogens. Antifungal lipopeptide genes were also screened in *Bacillus* spp. After lipopeptide treatment, live-dead staining with fluorescence microscopy along with bright-field and scanning electron microscopy (SEM) revealed structural deformation and cell death in *Fusarium* mycelia and spores. Furthermore, the development of pores in the membrane and leakages of protoplasmic substances from cells and ultimately death of hyphae and spores were also confirmed. In microcosm assays, treatment of seeds with *Bacillus subtilis* or application of its lipopeptide alone significantly protected seedlings from *Fusarium* sp. infection.

## Introduction

Plants have evolved with continuous interaction with diverse microorganisms. Many of these microorganisms actively colonize into the endospheric compartment of plants as endophytes and provide benefits to plants (Glassner et al., 2018; White et al., 2019). Endophytes, mostly bacteria and fungi, are frequently reported from all parts of the plant including root, stem, leaves, fruits, and seeds. Endophytes play an essential role in every stage of plant development and adaptation to various ecological conditions (Márquez et al., 2007; Llorens et al., 2019). In the recent past, many crop seeds including maize, wheat, rice, millets, cotton, etc., were reported to host endophytic bacteria (Gond et al., 2015; Herrera et al., 2016; Irizarry and White, 2017; Verma et al., 2017; Verma and White, 2018). Seed endophytic bacteria (SEB) are believed to have more influence on the development of plant because they can be transmitted to the next generation and become the first colonizers of roots and shoots of the seedlings after germination (Johnston-Monje and Raizada, 2011; Verma et al., 2019). Due to positional advantage, SEB may influence plant growth and fitness starting from seed germination to seedling formation and over time continue to influence plant development. SEB have been reported to increase the process of germination (Pitzschke, 2016), and plant growth by producing auxin, ethylene, mobilizing various nutrients (N, P, K, etc.) and producing siderophores (Ruiza et al., 2011; Verma and White, 2018; Maheshwari et al., 2019; Soldan et al., 2019; Kumar et al., 2020; Chang et al., 2021). Endophytes protect developing seedlings from soil pathogens by producing antimicrobial compounds; for example, *Bacillus* spp. produce antifungal lipopeptides including iturins, fengycins, surfactins, and bacillomycin whereas pseudomonads are known to produce antimicrobial metabolites like HCN, pyrrolnitrin and phenazine (Malfanova et al., 2012; Gond et al., 2015; Li et al., 2015; Verma and White, 2018). Endophytic bacteria also increase plant fitness indirectly by inducing or modulating plant gene expression related to growth development and defense (Gond et al., 2015; Mousa et al., 2016; Irizarry and White, 2018; Khalaf and Raizada, 2018). In comparison to other plant-associated microbes, SEB are more competent in benefiting hosts, but very little has been explored regarding their mechanisms of colonization during a seedling’s formation, or their functional roles in seedling development and protection. To the best of our knowledge, this is the first report which describes the role of pearl millet’s seed inhabiting bacteria on seedling establishment and protection. Pearl millet (*Pennisetum glaucum* L.) is an important, annual, small grain, warm season crop belonging to the family Poaceae, widely cultivated and consumed in tropical and subtropical countries of the world (Kumar et al., 2016). Pearl millet is a highly nutritious crop. Seeds contain high amounts of proteins, minerals (iron, zinc, sodium, phosphorus, and magnesium), vitamin B complexes, and high fiber (Kumar et al., 2016). In the present study, we hypothesized that seeds of pearl millet might be inhabited by endophytic bacteria that play a crucial role during the early development of seedlings, and also protect them from fungal pathogens. In this report, seven endophytic bacteria were isolated from pearl millet seeds and all the isolates were evaluated for their plant growth promoting and biocontrol activities. We found that the removal of bacteria from the seeds compromised the seedling’s development, and when we re-inoculated with the same bacteria, seedling development was restored. Colonization of SEB in root tissues was observed by fluorescent microscopy. In the seedling protection assay in microcosm, we found that treatments with *Bacillus subtilis* and its lipopeptides inhibited the growth of *Fusarium* and protected the seedlings from its infection. Using bright field, fluorescent and, scanning electron microscopy (SEM), we examined the lipopeptide effects on fungal hyphae and spores.

## Materials and Methods

### Plant Material

Pearl millet seeds were procured from B&B Organics Company, Tamil Nadu, India, and stored at 4°C in the refrigerator. Seeds were collected by B&B Organics Company from an agricultural field in Theni, Tamil Nadu (10.0104°N, 77.4768°E).

### Surface Sterilization and Disinfection of Seeds

Pearl millet seeds were surface sterilized by soaking seeds in 4% NaOCl solution for 10, 20, and 40 min. with constant shaking and after that, seeds were washed with sterile distilled water then transferred to 70% ethanol for one min. Then the seeds were washed several times with sterile distilled water. To check the efficacy of surface sterilization, 100 μl water from the last wash was transferred onto nutrient agar media. 10 min −4% NaOCl treatment was found efficient for surface sterilization. For complete disinfection of seeds, 10 min surface-sterilized seeds were dipped in streptomycin sulfate solution (100 μg ml^–1^) for different time periods including 6, 8, and 12 h; after that, seeds were washed thoroughly with sterile distilled water.

### Isolation, Molecular Identification and Phylogenetic Analysis of Seed Endophytic Bacteria Isolated From Pearl Millet Seeds

Around 100 surface-sterilized seeds of different time intervals (10, 20, and 40 min) were plotted onto the nutrient agar plates (8–10 seeds per plate) and incubated in a BOD incubator for 3–5 days at 27 ± 2°C. Bacterial colonies emerging around the seeds were sub-cultured. Based on growth pattern and color, bacterial isolates were selected and purified. All the purified isolates were preserved in 20% glycerol at −20°C freezer in the Department of Botany, BHU, Varanasi.

For molecular identification, all the pure bacterial isolates were grown in a nutrient broth with constant shaking with 200 rpm on a rotary shaker at 27 ± 2°C. Day-old bacterial cultures were used for genomic DNA extraction. For that, 1.5 ml bacterial cultures were centrifuged at 10,000 rpm for 2 min at 4°C and bacterial pellets were washed with de-ionized water to remove any metabolites. Bacterial genomic DNA was extracted by using Wizard^®^ Genomic DNA purification kit (Promega, United States) and the 16S rDNA conserved sequences were amplified using 16SF (5′-AGAGTTTGATCCTGGCTCAG-3′) and 16SR (5′-CTACGGCTACCTTGTTACGA-3′) primers. The PCR amplification was carried out by using T100 Thermal Cycler (Bio-Rad); the total volume of PCR reaction mixtures was 25 μl containing 12.5 μl of 2X PCR master mix (Promega, United States), 2 μl of 10 μM concentration of each primer, 5 μl DNA template (40 ng μl^–1^) and 3.5 μl nuclease free water. The following conditions were used for PCR amplification; initial denaturation 95°C for 5 min., followed by 35 cycles of denaturation step at 95°C for 1 min., annealing step at 55°C for 1 min. and extension step at 72°C for 1.5 min; final extension was at 72°C for 10 min. After PCR amplification, PCR products were visualized on 1% agarose gel with 1X TAE buffer. Purification and sequencing of amplified PCR products were done at AgriGenome Labs Pvt. Ltd. (Cochin, Kerala). The sequences were identified using BLASTn and closest matches were found by comparing the sequences with those in the NCBI GenBank database. Based on 16s rDNA sequences of SEB of pearl millet, the closest bacterial sequences were downloaded from the NCBI. All the nucleotide sequences were aligned by Clustal W and then the phylogenetic tree was constructed by the neighbor-joining (NJ) method using MEGA 11 software (Tamura et al., 2021). A bootstrap analysis was carried out with 1,000 repeats using the same software to test the phylogenetic tree’s reliability.

### Characterization of Seed Endophytic Bacteria for Plant Growth Promoting and Enzymatic Activities

Overnight bacterial cultures were streaked onto Pikovskaya agar media (Pikovskaya, 1948) and Aleksandrow agar media (Aleksandrov et al., 1967) and incubated for four days; and clear zone around the colonies was taken as confirmation of phosphate and potassium solubilization, respectively. Auxin (IAA) production was assessed by the method given by Gordon and Weber (1951). For that, all the bacterial isolates were grown in nutrient broth (5 g peptone, 1.5 g yeast extract, 1.5 g beef extract, 5 g sodium chloride, 1,000 ml distilled water) with or without tryptophan (100 μg ml^–1^). Four-day-old bacterial broth cultures were centrifuged at 4,500 rpm for 5 min. then 1 ml of culture supernatant and 2 ml of freshly prepared Salkowski reagent (1 ml of 0.5 mol l^–1^ FeCl_3_ was added to 50 ml sterile distilled water then finally mixed with 30 ml of H_2_SO_4_) were mixed together and incubated for 30 min., after which optical density (absorbance) was assessed at 530 nm using a spectrophotometer (Modal U-2900, Hitachi, Japan). IAA produced by endophytic bacteria was measured comparing with a standard curve as described elsewhere (Verma and White, 2018). Siderophore production test of bacterial isolates was performed by using chrome azurol S (CAS) agar plates (Schwyn and Neilands, 1987). Catalase activity was evaluated by using the tube slant method (MacFaddin, 2000). For that, 1 ml of 3% H_2_O_2_ was directly poured onto overnight grown bacterial cultures on nutrient agar slants and was placed over a dark background to observe bubble formation. For amylase activity, overnight bacterial cultures were streaked onto GYP agar plates (1 g glucose, 0.1 g yeast extract, 0.5 g peptone, 1.5% agar, 1,000 ml distilled water) supplemented with 1% starch (Hankin and Anagnostakis, 1975) and incubated for 5 days at 27 ± 2°C; after that, all plates were stained with iodine solution (1% iodine was mixed with 2% potassium iodide); the un-stained area around the bacterial isolate was measured. For cellulase activity, overnight bacterial cultures were streaked onto yeast extract peptone agar (yeast extract 0.1 g, peptone 0.5 g, agar 1.5%, distilled water 1,000 ml) containing 0.5% CMC (Na-carboxymethyl cellulose) (Teather and Wood, 1982). Five-day-old culture plates were stained with 0.2% (w/v) congo-red solution for 20 min and de-stained for 20 min with 1 M NaCl; the un-stained zone around the colony was measured. Pectinase activity of bacterial isolates was evaluated by the protocol described by Aguilar and Huitrón (1990). Bacterial isolates were inoculated onto pectin agar (5 g pectin, 1.5% agar, 1,000 ml distilled water) plates; after 5 days of incubation, plates were flooded with 1% (w/v) CTAB aqueous solution for 20 min. and cleared zones around colonies were observed to confirm pectinase activity. For protease activity, bacterial isolates were transferred onto GYP agar plates containing 0.4% gelatin (Sunitha et al., 2013) and after 5 days of incubation, saturated aqueous ammonium sulfate was flooded onto plates. The cleared zone around bacterial growth was measured. For chitinase activity, colloidal chitin was prepared by the method described by Hsu and Lockwood (1975). Chitin medium was prepared by adding 1% (w/v) colloidal chitin with 0.5% peptone, 0.1% yeast extract, and 1.5% agar in 80 ml sterile water and the final volume was maintained 100 ml. Overnight grown bacterial isolates were streaked on chitin plates and incubated for 5 days to check for clearing zones around colonies as positive indication of chitinase activity.

### Standardization of Growth Media for Re-inoculation Experiments

Several media including 1.5% agar, sand, filter paper, potting mix in magenta boxes including mixed substrates (cocopeat: perlite: sand) in two different ratio (2:1:1 and 2:2:1) were prepared. After that, various treatments including (1) completely disinfected seeds were (surface sterilized with 4% NaOCl and then treated with streptomycin sulfate-100 μg ml^–1^ for 8 h) then re-inoculated with selected SEB (10^6^–10^8^ cell ml^–1^) as treatments; (2) only surface-sterilized seeds as positive control, and (3) one set of completely disinfected seeds as negative control were transferred onto above mentioned growth media and incubated for 7–10 days at 27°C. All experiments were done in triplicate. The best medium was selected for further study.

### Re-inoculation Experiment With Selected Plant Growth Promoting Seed Endophytic Bacteria in Magenta Box

A potting mix containing cocopeat, perlite and sand in 2:2:1 ratio was found best out of all standardized media and selected for the re-inoculation experiment with SEB having plant growth promoting activities.

The experiment was set up as: (a) surface sterilized seeds with 4% NaOCl only (positive control-surface sterilized seeds), (b) surface sterilized seeds with 4% NaOCl + 100 μg ml^–1^ streptomycin sulfate (negative control-disinfected seeds) and (c) KAS1 (surface sterilized seeds with 4% NaOCl + 100 μg ml^–1^ streptomycin sulfate then treated with KAS1- *Kosakonia cowanii*) (d) KAS4 (surface sterilized seeds with 4% NaOCl + 100 μg ml^–1^ streptomycin sulfate then treated with KAS4–*Pantoea sterwarti*), and (e) KAS6 (surface sterilized seeds with 4% NaOCl + 100 μg ml^–1^ streptomycin sulfate then treated with KAS6–*Pseudomonas aeruginosa*). Each treatment was done in triplicate.

For re-inoculation of SEB, disinfected seeds (100–120) were treated with 5 ml suspensions of selected SEB (10^6^–10^8^ cell ml^–1^) separately for 2 h. Around 100 seeds were transferred into three magenta boxes containing potting mix for each treatment (25–30 seeds in each magenta box). All the magenta boxes were transferred in the plant growth chamber in controlled conditions (photoperiod 12 h, 80–90% humidity, and temp 27 ± 2°C). After 8 days, various growth parameters including root-shoot length, fresh weight, and photosynthetic pigments in leaves of seedlings were measured. Bacteria were re-isolated from roots of bacterial treated and control seedlings to prove that isolated bacteria from roots were the same or different than bacteria used during treatments with seeds. For that, roots were cut by sterile scalpel and transferred onto nutrient agar plates with the help of sterilized forceps and incubated in a BOD incubator for 2 days; afterward bacterial growth around roots was sub-cultured and identified by the molecular method as described earlier.

### Estimation of Photosynthetic Pigments

For quantification of photosynthetic pigments, 100 mg of leaf tissues from all treated seedlings were taken in a mortar (contained 15 ml of 80% acetone) and homogenized with a pestle; homogenized mixtures were filtered through a filter (0.45 μm) and absorbance was taken at 663, 645 and 440.5 nm by using UV/Visible spectrophotometer (Modal U-2900, Hitachi, Japan). The photosynthetic pigments including Chl a, b, and carotenoids were calculated by method given by Smith and Benitez (1955) using the following formulas:

Chlorophyll a mg g^–1^ fresh leaf tissue = 12.7 (OD) 663–2.69 (OD) 645 × (v/w × 1000),

Chlorophyll b mg g^–1^ fresh leaf tissue = 22.9 (OD) 645–4.68 (OD) 663 × (v/w × 1000),

Total carotenoids mg g^–1^ fresh leaf tissue = 46.95 (OD) 440.5–0.268 × chlorophyll (a + b).

### Microscopic Visualization of Seed Endophytic Bacteria Colonization on/in Root by Using Fluorescent Microscope

SYTO-9 fluorescent dye (Thermo Fisher Scientific, United States) was used for staining both live and dead bacteria which were present onto root parenchyma, root hairs, and inside the root tissues of seedlings. SYTO-9 stain was mixed in sterilized de-ionized water to make the final concentration up to 50 μM and incubated for 30 min. in dark condition at 27°C. Roots from both control and treated seedlings were collected from magenta boxes and cleaned with sterile water. 1–2 cm length size of root was cut by sterilized scalpel and was transferred on the slide then 20 μl (50 μM) of SYTO 9 solution was poured on the root surfaces and incubated for 5 min. in dark conditions after which roots were examined under a fluorescent microscope (Nikon, Japan).

### Antifungal Activity of Endophytic Bacterial Isolates

All the bacterial isolates from pearl millet seeds were screened for antagonistic activity against selected fungal phytopathogens including *Fusarium* sp., *Curvularia* sp., *Alternaria* sp., *Rhizoctonia solani*, *Epicoccum sorghinum*, and *Exserohilum rostratum* onto potato dextrose agar media using dual culture technique. After 5 days of incubation in a BOD incubator at 27°C, fungal growth inhibition (%) due to antagonistic bacteria was calculated by the formula described by Whipps (1997):

% inhibition of fungal growth = R1–R2/R1 × 100,

where R1 is the growth of pathogenic fungi on the control plate and R2 is the growth of pathogenic fungi toward the antagonistic bacterial isolates on test plates.

### Screening of Lipopeptide Genes in Bacterial Isolates

All the bacterial isolates were screened for selected lipopeptide genes including surfactin, bacillomycin D, iturin A, and fengycin using primers mentioned in Supplementary Table 3. The PCR amplification reaction was set up as initial denaturation (5 min at 95°C), followed by 35 cycles of denaturation (45 s at 95°C), annealing (1 min for 55°C), extension (1 min for 72°C), and final extension for 10 min at 72°C. Amplified PCR products were sent for purification and gene-specific DNA sequencing; obtained DNA sequences were identified using the BLASTn program to confirm lipopeptide genes present in respective bacterial isolates.

### Lipopeptide Extraction and Antifungal Disc Diffusion Assay

Based on the presence of lipopeptide genes in bacterial isolates, three bacterial isolates (*Bacillus subtilis*-KAS-2, *Bacillus tequilensis*-KAS3, and *Bacillus velezensis*-KAS7) were grown in liquid culture for lipopeptide production and extraction. Using the method described by Gond et al. (2015), lipopeptide was extracted from bacterial isolates. For that, selected bacterial isolates (KAS2, KAS3, and KAS7) were grown in nutrient broth with constant shaking with 200 rpm on a rotary shaker at 27°C. After 4 days of incubation, bacterial cultures were centrifuged at 4,500 rpm (15 min at 4°C); culture supernatants were collected and acidified (up to pH 2°C) with concentrated HCl and were incubated overnight at 4°C. Acidified culture supernatants were centrifuged at 9,000 rpm (15 min at 4°C). The pellets were collected and dissolved in methanol and then filtered through a 0.22 μm filter membrane to remove bacterial cells debris. Methanol filtrate was dried through a vacuum evaporator and stored at 4°C. Using disc diffusion assays, the antifungal activity of lipopeptide was checked against fungal phytopathogens including *Fusarium* sp., *Curvularia* sp., *Alternaria* sp., *Rhizoctonia solani*, *Epicoccum sorghinum*, and *Exserohilum rostratum*. For that, a sterile paper disc was loaded with 20 μl (200 μg) of methanolic solution of lipopeptides and the control disc contained only 20 μl methanol. Loaded discs were transferred onto potato dextrose agar (PDA) plates with a centrally placed small disc of fungal mycelia and incubated for 4 days. Stereo microscopy (Magnus, India) was used to observe the zone of inhibition between lipopeptide-loaded disc and fungal pathogens.

### Effects of Different Concentrations of Lipopeptides on *Fusarium* sp. Growth and Evaluation of Minimum Inhibitory Concentration

Different concentrations (0.25, 0.50, 1, 2, and 3 mg ml^–l^) of lipopeptide extracts (from *Bacillus subtilis*) were prepared in PDA media. A 5 mm diameter fungal mycelial disc was placed in the center onto PDA plate and incubated in a BOD incubator for 4 days. Growth inhibition of the fungus was measured using the following formula (Borah et al., 2016):

Mycelial growth inhibition = 100–(Diameter of mycelium growth in lipopeptide medium/Diameter of mycelium growth on control medium plate × 100).

For calculation of MIC, several concentrations (0.25, 0.50, 1, 2, 3, 4, 5, 6, 7, and 8 mg ml^–1^) of lipopeptide extract (from *Bacillus subtilis*) were prepared in potato dextrose broth (PDB) medium in test tubes having 10 ml each concentration. 50 μl of fungal spore suspension (10^3^–10^4^ spores ml^–1^) were transferred to each test tube and incubated for 7 days. After 7 days of incubation, fungal growth was checked for MIC.

### Microscopic Analysis of the Effect of *Bacillus subtilis* and Its Lipopeptides on *Fusarium* Hyphal and Spore Structure

Effects of treatment with both *Bacillus subtilis* and its lipopeptide extract on *Fusarium* hyphal and spore structures were examined under bright field, fluorescent, and SEM. Lactophenol cotton blue (Himedia) was used for observation of fungal mycelia and spores under light microscopy (Olympus, India). Fluorescent stains such as SYTO-9 (Thermo Fisher Scientific, United States), propidium iodide (Sigma-Aldrich), DAPI (Sigma-Aldrich), and calcofluor white (Sigma-Aldrich) were used to study the fungal mycelia and spores under fluorescent microscopy (Nikon, Japan). For SEM, sample preparation was done using the method described by Wu et al. (2019). A portion of fungal mycelia was placed onto the slide and fixed with 2.5% glutaraldehyde. After 2 days of incubation, it was rinsed with phosphate buffer (10 mM) and dehydrated with increasing concentrations of ethanol. Dehydrated fungal samples were examined under SEM (EVO 18, Carl Zeiss, Germany) in the Department of Geology, BHU, Varanasi.

### Preparation of Fluorescent Stains

Twenty-five micromolar concentration of SYTO 9, propidium iodide (PI), DAPI and calcofluor white (CFW) were prepared separately from their respective stock solutions. A combination of SYTO 9-PI, DAPI-PI, and CFW-PI were prepared in a 1:1 ratio and 20 μl from all combination of stains were used separately for fungal staining. After putting the combination of stains on mycelial pieces on a slide, samples were incubated for 5 min. in a dark room at room temperature before being examined under a fluorescent microscope. All selected fungal tissues were observed using fluorescent microscopy (Nikon DAPI-FITC-TRITC filter combinations).

### Seedling Protection Assay in Microcosms

In seedling protection assays, surface-sterilized seeds (4% NaOCl–10 min) were treated with *Bacillus subtilis* (10^5^–10^7^ cells ml^–1^) and its lipopeptides (200 μg ml^–1^) separately then inoculated with *Fusarium* sp. spores (10^3^–10^4^ spores ml^–1^). Seeds only inoculated with fungal spores were set up as control. From all treatments, 20–25 seeds were transferred into each magenta box, containing sterile cocopeat, perlite and sand in a 2:2:1 ratio, then magenta boxes were placed into a growth chamber. Each treatment was done in triplicate.

### Statistical Analysis

Microsoft excel was used for the preparation of bar diagrams and measurement of standard errors. SPSS-16 program was used for one-way ANOVA followed by *post hoc* Duncan analysis to evaluate the significant difference in means of root-shoot length, fresh weights and photosynthetic pigments among and between the treatments and controls.

## Results

### Seed Surface Sterilization, Disinfection, Isolation and Identification of Seed Endophytic Bacteria

Surface sterilization with 4% sodium hypochlorite for 10 min. followed by 1 min. in 70% ethanol was found effective since no bacteria emerged from the last wash solution of the treatment. A total of seven types of endophytic bacteria were isolated from pearl millet seeds on nutrient agar media. Five bacterial isolates (KAS1, KAS2, KAS3, KAS4, and KAS5), three (KAS4, KAS5, and KAS7), and two (KAS6 and KAS7) were isolated from 10, 20, and 40 min. surface-sterilized seeds with sodium hypochlorite, respectively. KAS1 and KAS4 were found to be the most common isolates with 65 and 21% colonization in seeds (Table 1). No bacteria observed around completely disinfected (treated with sodium hypochlorite-10 min + 100 μg ml^–1^ streptomycin −8 h) seeds on the nutrient agar plate. Completely disinfected seeds were treated as endophytes-free and were further used for the re-inoculation experiment.

**TABLE 1:** List of molecularly identified endophytic bacteria using 16S rDNA sequencing with their closet matches, percentage similarity and accession no. of isolates.

Bacterial isolates and% CF	Closet matches	Similarity (%)	GenBank accession no. of bacterial isolates
KAS1 (65.55)	*Kosakonia cowanii*	99.65	MN134077
KAS2 (6.66)	*Bacillus subtilis*	100	MN367975
KAS3 (6.66)	*Bacillus tequilensis*	100	MN134078
KAS4 (21.11)	*Pantoea stewartii*	100	MN134079
KAS5 (3.33)	*Paenibacillus dendritiformis*	99.66	MN134080
KAS6 (3.33)	*Pseudomonas aeruginosa*	99.89	MN134081
KAS7 (1.11)	*Bacillus velezensis*	100	MN134082

*% CF–Colonization Frequency of bacterial isolates in seeds.*

The bacterial isolates were identified as KAS1, KAS2, KAS3, KAS4, KAS5, KAS6, and KAS7 (Table 1). The phylogenetic analysis confirmed close relationship of bacterial isolates of pearl millet seed with the corresponding species recovered from the NCBI database (Supplementary Figure 1).

### Plant Growth Promoting and Enzymatic Activities of Seed Endophytic Bacteria

Bacterial isolates (KAS1, KAS4, and KAS6) were found to produce IAA (auxin) in greater amounts as compared to KAS2, KAS3, KAS5, and KAS7 isolates in nutrient broth (both with or without tryptophan supplementation) (Table 2). Bacterial isolates, KAS1 and KAS4, showed very good phosphate and potassium solubilization activities while three bacterial isolates: KAS2, KAS4, and KAS7, showed siderophore production activity (Table 2). *Bacillus* strains: KAS2, KAS3, and KAS7, were found to be more active in amylase and cellulase production activities. Out of seven strains, only KAS2 showed pectinase activity and KAS5 showed chitinase activity (Supplementary Table 2). Except for KAS5, all the isolates: KAS1, KAS2, KAS3, KAS4, KAS6, and KAS7, showed catalase activity (Supplementary Figure 2). Except for KAS1, all isolates: KAS2, KAS3, KAS4, KAS5, KAS6, and KAS7, showed protease activity (Supplementary Table 2).

**TABLE 2:** Auxin (IAA) production, phosphate solubilization, potassium solubilization, and siderophore production activities of seed endophytic bacteria.

Bacterial isolates	Auxin production (μg ml^–1^ ± SE)		Phosphate soulubilization	Potassium soulubilization	Siderophore
	Without Trp	With Trp			
KAS1	7.2 ± 0.06	9.0 ± 0.09	+++	++	–
KAS2	3.2 ± 0.08	4.0 ± 0.10	+	–	++
KAS3	2.2 ± 0.11	3.3 ± 0.11	+	–	–
KAS4	7.7 ± 0.26	13.9 ± 0.36	+++	++	+
KAS5	2.3 ± 0.08	3.2 ± 0.13	–	–	–
KAS6	4.9 ± 0.18	6.2 ± 012	++	–	–
KAS7	2.0 ± 0.13	3.5 ± 0.10	–	–	+++

*Where, (-, no activity; +, <5 mm clear zone; ++, 5–10 mm clear zone; +++, more than 10 mm clear zone around endophytic bacterial isolates); KAS1, Kosakonia cowanii, KAS2, Bacillus subtilis, KAS3, Bacillus tequilensis, KAS4, Pantoea stewartii, KAS5, Paenibacillus dendritiformis, KAS6, Pseudomonas aeroginosa, KAS7, Bacillus velezensis, (Trp, Tryptophan).*

### Standardization of Growth Media for Re-inoculation Experiments

Out of several media (including 1.5% agar, filter paper, river sand, a potting mix including ratio cocopeat: perlite: sand−2:1:1 and ratio −2:2:1) which was used for initial standardization, a potting mix containing cocopeat, perlite and sand with 2:2:1 ratio showed best result of seed germination and seedling development compare to others, hence this medium was further selected for re-inoculation experiments.

### Removal and Re-inoculation of Seed Endophytic Bacteria on Seedling Growth and Development

**FIGURE 1:**
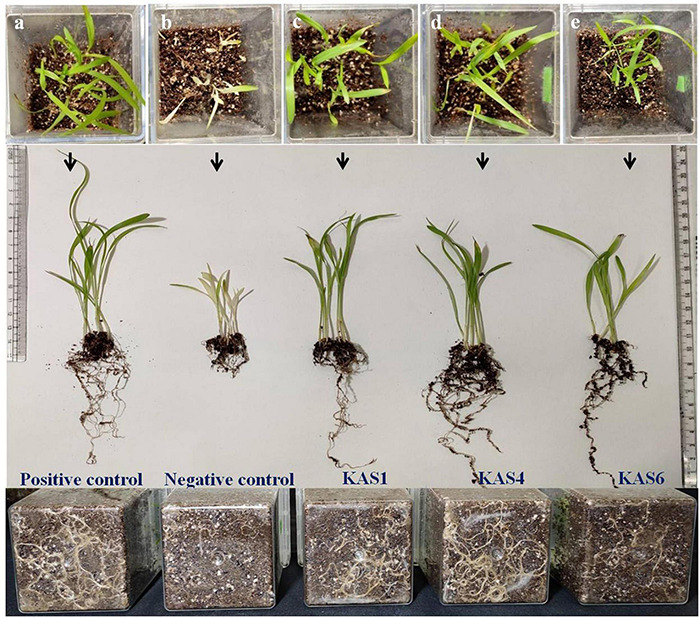
Effect of disinfection and re-inoculation of seed endophytic bacteria (SEB) on seedling growth and root-shoot length (seedlings were 8 days old). **(a)** Positive control is where seeds were treated only with NaOCl; **(b)** negative control is where seeds were treated with NaOCl + antibiotic; **(c–e)** KAS1, KAS4, and KAS6 are where seeds initially treated NaOCl + antibiotic were inoculated with strains KAS1 (*Kosakonia cowanii*), KAS4 (*Pantoea stewartii*), and KAS6 (*Pseudomonas aeruginosa*), respectively. In the first lane: seedlings grown in magenta boxes viewed from the top; second lane: seedlings taken out from the boxes and viewed; and third lane: viewed from the back of magenta boxes showing root growth.

**FIGURE 2:**
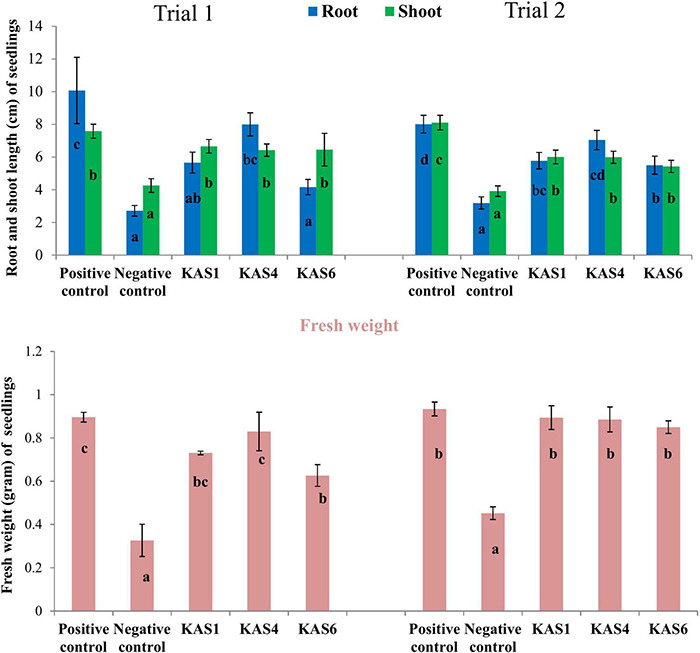
Effect of removal and re-inoculation of SEB on the root-shoot length and fresh weight of seedlings. Positive control: where seeds were treated only with NaOCl; Negative control: where seeds were treated with NaOCl + antibiotic; KAS1, KAS4, and KAS6: where seeds initially treated NaOCl + antibiotic then inoculated with strains KAS1, KAS4, and KAS6. Different letters represent the significant differences among the means of treatments (*N* = 30, *P* ≤ 0.05). Experiment was repeated in two trials (Trial 1 and Trial 2).

**FIGURE 3:**
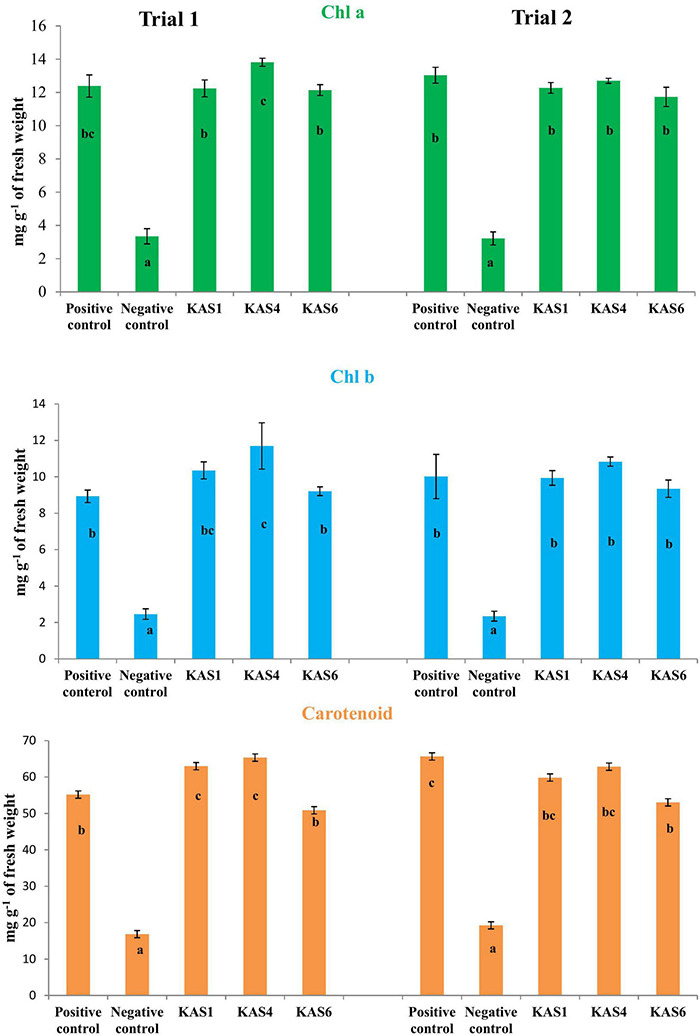
Effect of removal and re-inoculation of SEB on the content of photosynthetic pigments of leaf tissues from 8 day old seedlings. First lane- Chlorophyll a, second lane- chlorophyll b and third lane- carotenoids. Positive control: seeds were treated only with NaOCl; negative control: seeds were treated with NaOCl + antibiotic; KAS1, KAS4, and KAS6: seeds initially treated with NaOCl + antibiotic and then inoculated with strains KAS1 (*Kosakonia cowanii*), KAS4 (*Pantoea stewartii*), and KAS6 (*Pseudomonas aeruginosa*). Different letters represent the significant differences among the means of treatments. (*P* ≤ 005). Experiment was repeated in two trials (Trial 1 and Trial 2).

Based on plant growth promoting activities, the best three SEB, including KAS1, KAS4, and KAS6, were used for re-inoculation experiments with disinfected seeds. All the bacterial isolates including KAS1, KAS4, and KAS6 significantly increased the root-shoot length and fresh weight of seedlings as compared to the negative control (without bacteria) (Figures 1, 2); further, re-inoculation with bacteria improved chlorophylls a and b, and carotenoids contents in the leaves of seedlings (Figure 3). Out of the three, KAS4 was found to be best in stimulating seedling development in terms of root-shoot length and photosynthetic pigments compare to negative controls (Figures 1–3). We repeated the experiment twice (trials 1 and 2) and found similar trends in the results. We also re-isolated bacteria from the roots of treated seedlings and confirmed their identities (Supplementary Table 1).

### Microscopic Visualization of Roots for Colonization of Seed Endophytic Bacteria

**FIGURE 4:**
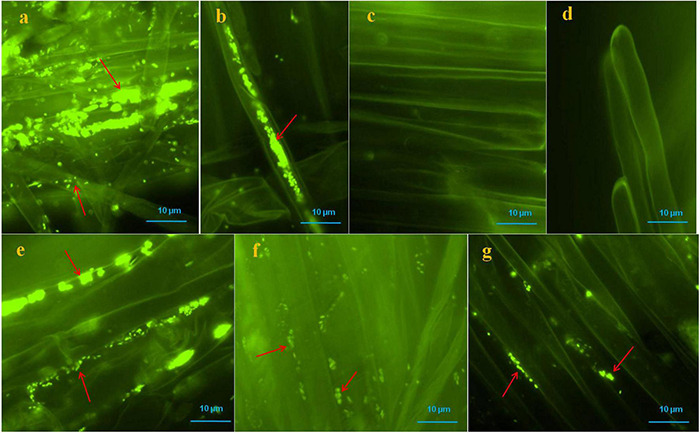
Microscopic visualization of bacteria on or in roots of pearl millet seedlings after SYTO-9 staining under fluorescence microscope. Where, in **(a,b)** several bacteria were observed on the root surfaces and inside of the root parenchyma and hair cells of positive control seedlings (arrows); **(c,d)** no bacteria were observed onto root surfaces and root hairs of negative control seedlings; **(e–g)** KAS1 (*Kosakonia cowanii*), KAS4 (*Pantoea stewartii*), and KAS6 (*Pseudomonas aeruginosa*) were observed on surfaces of root and in intercellular spaces of root parenchyma cells, respectively (arrows).

Colonization of bacteria was observed on the root surface, root hairs and, inter- and intra-cellular spaces of root parenchyma cells of positive controls and bacterial treated seedlings which were visible with SYTO 9 stain under fluorescent microscopy, however, no bacteria were found on the root surface, and root hairs of negative control seedlings (Figure 4). Multiple roots from each sample were visualized and we found more or less similar colonization patterns.

### Antifungal Activity of Endophytic Bacterial Isolates

Out of seven bacterial isolates tested, five bacterial isolates:KAS2, KAS3, KAS4, KAS6, and KAS7, showed significant antifungal activity against all the tested fungal pathogens including *Fusarium* sp., *Curvularia* sp., *Alternaria* sp., *Rhizoctonia solani*, *Epicoccum sorghinum*, and *Exserohilum rostratum* (Table 3).

**TABLE 3:** Antagonistic activity (as% inhibition) of endophytic bacteria against selected fungal phytopathogens in dual plate culture.

Bacterial isolates	*Fusarium* sp.	*Rhizoctonia solani*	*Alternaria* sp.	*Curvularia* sp.	*Eppicocum sorghinum*	*Exserohilum rostratum*
KAS1	66.7	26.1	65	0	21.4	20
KAS2	50	39.1	70	65	50	56
KAS3	43.33	37	65	40	46.4	56
KAS4	60	23.9	65	28	42.8	66
KAS5	25	4.3	0	0	0	0
KAS6	80	50	70	80	71.4	80
KAS7	50	43.5	65	70	65	66

*Where, KAS1, Kosakonia cowanii, KAS2, Bacillus subtilis, KAS3, Bacillus tequilensis, KAS4, Pantoea stewartii, KAS5, Paenibacillus dendritiformis, KAS6, Pseudomonas aeroginosa, KAS7, Bacillus velezensis.*

### Screening of Lipopeptide Genes and Antifungal Activity of Lipopeptide

Three *Bacillus* spp. including KAS2, KAS3, and KAS7, which were most active against fungal phytopathogens were also found to have at least one antifungal lipopeptide gene (Supplementary Table 4). Gene-specific sequencing (lipopeptide genes) also confirmed the lipopeptide genes present in specific *Bacillus* spp. (Supplementary Table 4). Furthermore, in disc diffusion assays, lipopeptide extracted from KAS2, KAS3, and KAS7, showed good antifungal activity against pathogenic fungi, including *Fusarium* sp., *Curvularia* sp., *Alternaria* sp., *Rhizoctonia solani*, *Epicoccum sorghinum*, and *Exserohilum rostratum* (Supplementary Figure 3).

### Effects of Different Concentrations of Lipopeptide on *Fusarium* sp. and Minimum Inhibitory Concentration

**FIGURE 5:**
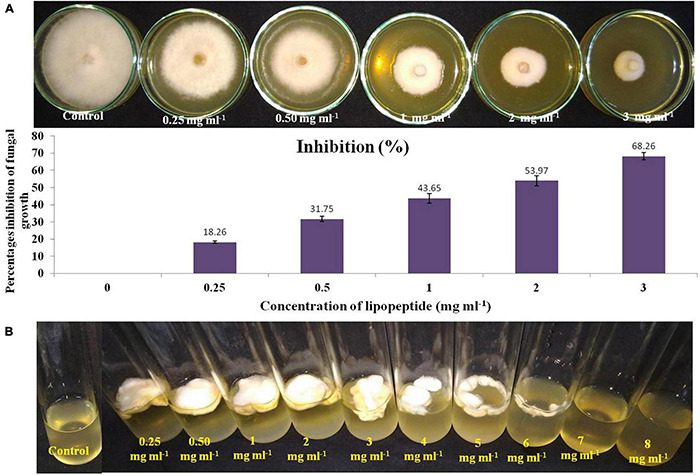
Measurement of minimum inhibitory concentration (MIC) of lipopeptide against *Fusarium* sp. **(A)**. Effects of different concentration of lipopeptides on PDA plate (A-first lane) and their percentages inhibition (A-second lane), **(B)**. Effects of different concentration of lipopeptides in PDB, (Control A- without lipopeptide, Control B- without lipopeptide and fungus).

To check the effective concentration of lipopeptide for antifungal activity against *Fusarium* sp., the fungus was challenged with different concentrations of lipopeptide prepared in PDA as well as PDB. With increasing concentrations of lipopeptides, growth of *Fusarium* was found reduced and stopped in broth (PDB) and PDA plates (Figure 5). After 4 days of incubation, more than 50% of fungal growth was inhibited at 2 mg ml^–1^ on PDA media. No fungal growth was observed in PDB at 7 mg ml^–1^ concentration of lipopeptide after 7 days of incubation. Hence, 7 mg ml^–1^ concentration of lipopeptide was recorded as MIC of lipopeptide against *Fusarium* sp. (Figure 5).

### Microscopic Analysis of Effects of *Bacillus subtilis* and Its Lipopeptides on *Fusarium* Mycelia and Spores

**FIGURE 6:**
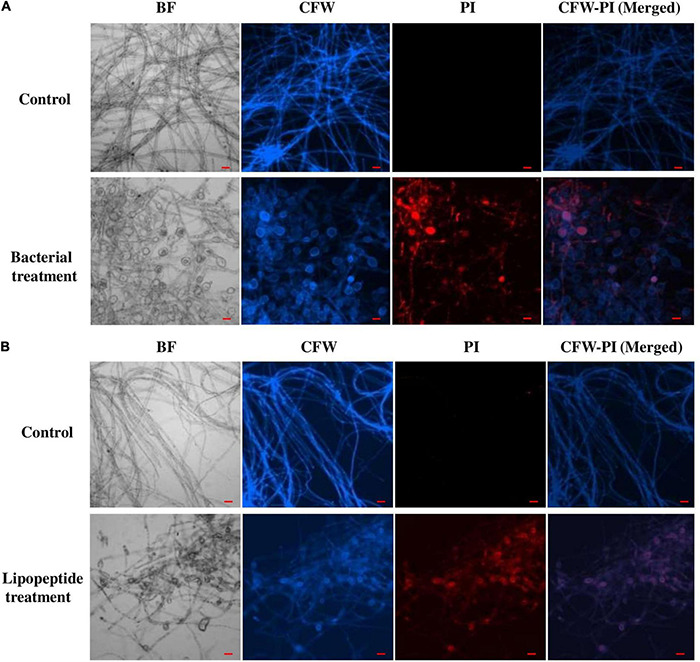
Effect of bacterial (*Bacillus subtilis*) **(A)** and lipopeptides **(B)** on *Fusarium* hyphae was observed under bright field and fluorescent microscopy (CFW and PI). Bacterial treatment was done by co-cultivation of *Fusarium* with *Bacillus subtilis* in mixed media of nutrient and potato dextrose agar. Lipopeptide treatment was done by disc diffusion (each disc contain 20 μl of 10 μg μl^– 1^ methanolic solution of lipopeptide), hyphae growing near the disc was taken for microscopy. In control-A fungus was not challenged with bacteria and control B- disc was only loaded with 20 μl of methanol without lipopeptide (Scale bar = 20 μm).

**FIGURE 7:**
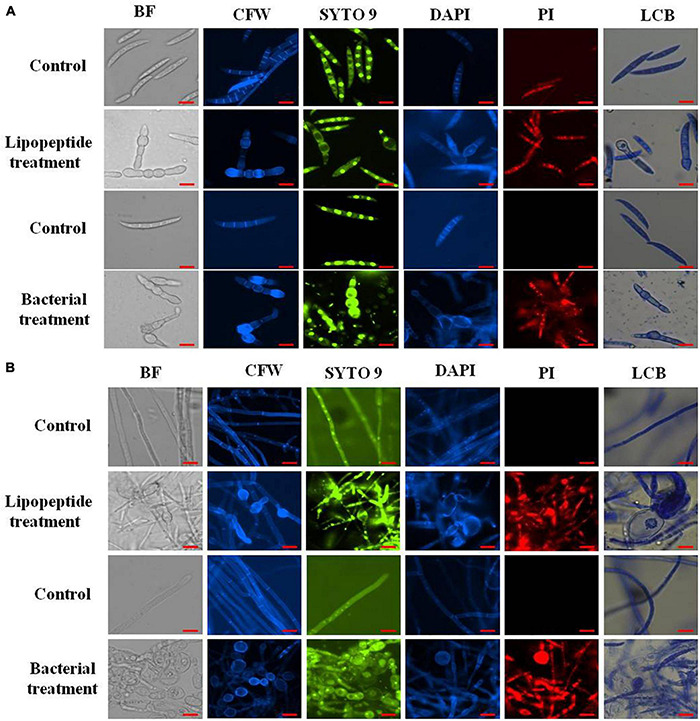
Closer view of effect of bacterial (*Bacillus subtilis*) and lipopeptides on fungal spores **(A)** and hyphae **(B)** of *Fusarium* sp. under bright field, fluorescent microscopy (CFW, Syto-9, DAPI, and PI) and lactophenol cotton blue (LCB) light microscopy. Bacterial treatment was done by co-cultivation of *Fusarium* with *Bacillus subtilis* in mixed media of nutrient and potato dextrose agar/broth. Lipopeptide treatment was done by disc diffusion (each disc contain 20 μl of 10 μg μl^–1^ methanolic solution of lipopeptide), hyphae and spores growing near the disc was taken for microscopy. In control-A fungus was not challenged with bacteria and control B- disc was only loaded with 20 μl of methanol without lipopeptide (Scale bar = 10 μm).

**FIGURE 8:**
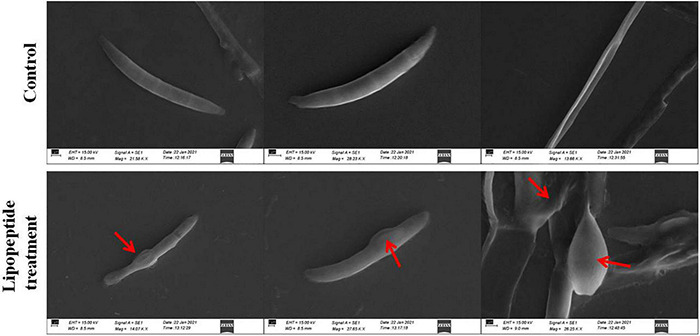
Effects of lipopeptide on fungal hyphae and spores were observed under scanning electron microscopy (SEM). Lipopeptide treatment was done as described in Figures 6, 7. In control spores and hyphae were found intact and normal in structure whereas in treatment, deformation, swelling and rupturing of spores and hyphae were observed (arrows).

*Bacillus subtilis* and its lipopeptide extract showed significant inhibitory effect on the tested fungal pathogen; it retarded the growth and also caused deformation in hyphal and spore structures of the fungus. Microscopic analysis with bright field, fluorescence, and SEM of bacterial and lipopeptide treated *Fusarium* revealed the development of abnormal structures in fungal hyphae and spores including a ball like swelling and bulging. Furthermore, disintegration and lysis of spore/cells were also observed. However, smooth and normal structures of hyphae and spores (without any deformation) were observed in untreated (control) (Figures 6–8). In propidium iodide (PI)

staining (which stains only dead cells), significant numbers of dead cells of *Fusarium* hyphae and their spores were observed in *Bacillus* and lipopeptide treatments, however, no or very few damaged or dead cells were observed in controls. Lactophenol cotton blue (LCB), SYTO-9, CFW, and DAPI stained both live and dead cells. PI in combination with other stains (SYTO-9, DAPI, and CFW) helped in improving visualization and differentiation between the dead and living structures of fungal hyphae and spores. Live-dead staining with combination of PI and other stains (SYTO-9, DAPI, and CFW) clearly showed that the burst and dead parts of hyphae and spores were only stained with PI, while CFW and other stains, stained all parts of hyphae and spores (Figures 6, 7). CFW also differentiated between dead and live cells by staining the chitin present in the cell wall of hyphae and spores. Live fungal mycelial cells and spores were clearly visible from tips and margins (intact cell walls) because CFW stained the chitin present in the cell wall and spores while burst/ruptured or damaged cells were not clearly visible (blurred) at tips and margins of spores and mycelial cells (Figures 6, 7).

### Seedling Protection From *Fusarium* Infection

**FIGURE 9:**
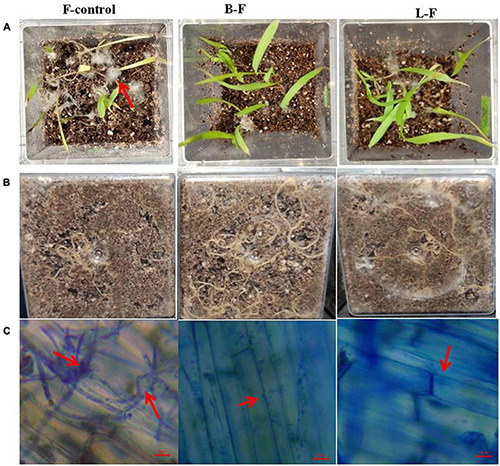
Effect of bacterium and its lipopeptide treatments in protection of seedlings from *Fusarium* sp. infection inmicrocosm assay in magenta boxes. Where, surface sterilized seeds were inoculated with fungal spores only (column 1: F), *Bacillus subtilis* and fungal spores (column 2: B-F) and lipopeptide and fungal spores (column 3:L-F). Lane **(A)** are the images of 8 day old seedlings from the top of the magenta boxes, lane **(B)** image from the bottom of the same magenta boxes showing root growth, and lane **(C)**- microscopic images of root surface from all corresponding treatment showing heavy infection of fungal pathogen in control in comparison to treatments (arrows).

Seeds treated with the bacterium *Bacillus subtilis* and its lipopeptides grew as healthy seedlings with few or no infections when challenged with *Fusarium* spores, but untreated control seedlings were heavily infected and almost all seedlings were collapsed within 8–10 days of infection due to the pathogen (Figure 9). Microscopy of the roots also showed that untreated control seedling roots were extensively colonized by the fungus, whereas very little or no infection was observed in treatments.

## Discussion

### Surface Sterilization, Isolation, and Removal of Seed Endophytic Bacteria From Pearl Millet Seeds

All plants and their tissues contain multiple microbes internally that play crucial roles in plant development and protection against diseases. Past studies have shown that seed-associated microbes, including bacteria and fungi, improve seed germination and seedling establishment (Puente et al., 2009; Rasmussen et al., 2015; Cope-Selby et al., 2017; Irizarry and White, 2017; Verma et al., 2017). Many crop seeds, including maize, rice, wheat, and cotton, have been reported to harbor bacterial seed endophytes that have seedling growth modulation activity (Gond et al., 2015; Pitzschke, 2016; Irizarry and White, 2017; Verma et al., 2017). In the present study, we found that a 10 min. sodium hypochlorite treatment followed by 70% ethanol for 1 min. was efficient to completely remove surface microflora since no growth of bacteria was observed from the last wash solution on NA plates (Kumar et al., 2020). As we increased the sterilization time from 10 to 20 and 40 min., numbers of recovery of isolates were reduced, and seeds were also found to have reduced germination frequency, which could be due to over-sterilization. Overall, seven endophytic bacteria, including KAS1, KAS2, KAS3, KAS4, KAS5, KAS6, and KAS7, were isolated from the surface-sterilized seeds and identified by using 16S rDNA sequencing (Table 1). In this study, KAS1 was found to be the most dominant isolate since it was most frequently isolated from seeds of millet followed by KAS4 (Table 1). Recently, a study reported the occurrence of *Kosakonia cowanii* as the most common SEB in seeds of *Lactuca serriola* (Jeong et al., 2021). *Pantoea* and *Bacillus* spp. were also recovered as seed endophytes in previous studies (Pitzschke, 2016; Irizarry and White, 2017; Verma and White, 2018). We successfully disinfected seeds (free from SEB) by further treating NaOCl-surface sterilized seeds with streptomycin sulfate (100 μg ml^–1^) for 8 h and disinfection was confirmed by non-emergence of bacteria from the seeds onto NA plates. Application of antibiotics with standardized concentrations and time has been suggested in the past for curing the seeds for re-inoculation experiments (Puente et al., 2009; Verma et al., 2017). However, the possibilities of non-culturable bacteria present inside the seeds have not been ruled out (Hardoim et al., 2012; Truyens et al., 2015; Chimwamurombe et al., 2016; Pei et al., 2017).

### Crucial Role of Seed Endophytic Bacteria in Seedling Growth and Development

Seedling growth and development was found suppressed in terms of root-shoot length and fresh biomass when seeds were cured using antibiotic (negative control) in comparison to seeds carrying their natural SEB (positive control) (Figures 1, 2). We also found that seedlings developed from cleaned seeds started showing bleaching effects within 4–5 days of their growth which was further evidenced by reduced photosynthetic pigments in comparison to the SEB-positive controls (Figure 3). This demonstrates the importance of the seed-vectored endophytic bacteria during the early stages of seedling development. Research in the past has shown that the removal of bacteria from seeds negatively affects germination and seedling development (Puente et al., 2009; Holland, 2016; Verma and White, 2018). Puente et al. (2009) in their study found that elimination of bacteria from seeds using antibiotics significantly suppressed seedling development in cardon cactus plants. Holland (2016) reported that the removal of bacteria from several crop seeds including soybean, rice, and kidney beans significantly reduced germination and further seedling development.

When we re-inoculated the disinfected seeds with their own best plant-growth promoting SEB (Table 2), including KAS1, KAS4, and KAS6, seedlings showed restored growth and development in terms of root-shoot lengths and fresh weights (Figures 1, 2), and improved photosynthetic pigments, such as chlorophyll a, b, and carotenoids in their leaves (Figure 3). We found similar effects in two experiment trials. Seed endophytes are primary symbionts that may play an essential role in germination and seedling development (Puente et al., 2009; Herrera et al., 2016; Chen et al., 2017; Ridout et al., 2019; Kumar et al., 2020). In this study, all seven isolated SEB showed IAA production, five: KAS1, KAS2, KAS3, KAS4, and KAS6, showed P solubilization and two: KAS1 and KAS 4, showed K solubilization activities (Table 2), and this could be a reason behind the positive effect of SEB on seedling growth restoration (Etesami et al., 2017; Afzal et al., 2019; Abdelaal et al., 2021). SEB KAS2, KAS4, and KAS7, also showed siderophore production activity. Siderophores are iron-chelating agents that have a greater affinity for ferric ions and convert insoluble ferric form of iron to soluble ferrous form which are easily accessible to the plant. Siderophore producing bacteria also reduce the growth of pathogenic fungi by reducing the iron availability in soil (Afzal et al., 2019; Duponnois and L’Hoir, 2021; Kumar et al., 2021). In both trials, we found that isolates KAS1, and KAS4 significantly increased the root-shoot lengths, biomass, and photosynthetic pigments in seedlings, and both isolates were the highest producers of IAA and the best P solubilizers (Table 2). *Kosakonia cowanii* and *Pantoea* species have been reported as seed vectored bacteria in many crop seeds with plant-growth promoting activities (Chimwamurombe et al., 2016; Verma et al., 2017; Jeong et al., 2021). The profound effect of KAS1 and KAS4 treatment on root architecture development of seedlings may be because of their IAA activities. IAA is known to increase root architecture in plants and endophyte-mediated IAA effects on root development have been described in the past (Maggini et al., 2019; Verma et al., 2021). A study of the interaction of *Arabidopsis* seedlings with *Pseudomonas* significantly improved the root structure, and further study suggested that bacterium-mediated auxin signaling is important for better development of root system architecture (Zamioudis et al., 2015). Endophytes may also modulate the endogenous level of auxin in their host plants (Wang et al., 2015; Maggini et al., 2019). Recently, Chang et al. (2021) reported that root hair elongation may be modulated by ethylene secreted by SEB that become intracellular in root cells; these intracellular microbes were also found to secrete nitric oxide. Both ethylene and nitric oxide together could function with auxin to modulate development in seedlings. Regardless of the exact mechanism, SEB KAS1, KAS4, and KAS6 of pearl millet were shown to have an important role in seedling development.

### Colonization of Seed Endophytic Bacteria Into Root Tissue of Seedlings and Proposed Nutrient Mobilization

In our microscopy study of treated seedling roots, we observed that KAS1, KAS4, and KAS6 colonized into inter and intracellular spaces of root parenchyma and root hairs cells (Figures 4e–g). In positive controls (with all possible SEB), SEB colonized inside root hairs and on and in root parenchyma cells (Figures 4a,b). No bacteria were found in the roots of negative control seedlings (Figures 4c,d). We also re-isolated the bacteria from the roots of treated seedlings and confirmed that they were identical to the inoculated bacteria by 16S rDNA sequencing (Supplementary Table 1). Colonization and growth of seed inhabiting bacteria onto developing roots and rhizosphere confirmed the importance of SEB in developing rhizospheric microbiota (López-López et al., 2010; Truyens et al., 2015; Verma et al., 2019). Successful colonization of SEB onto root surfaces and rhizospheres may help developing seedlings in nutrient acquisition in two ways; first, they may mineralize/solubilize nutrients in the rhizosphere, and these will be easily available to root hairs; secondly, within root parenchyma and root hair cells, nutrients may be extracted from bacteria oxidatively through the rhizophagy cycle (Paungfoo-Lonhienne et al., 2010; White et al., 2018). It has been shown that the secretion of ethylene by bacteria, triggers release of superoxide by the root cell, and it is hypothesized that superoxide produced by root cells acts on bacteria in extraction of nutrients from cell walls and cytoplasm (White et al., 2018; Chang et al., 2021; Verma et al., 2021). In this study, most of the isolates showed at least one extracellular enzyme production activity including cellulase, pectinase and amylase. Hydrolytic enzymes like cellulase, pectinase and amylase might play important role in endophytic colonization and establishment of bacteria in plant tissues (Dogan and Taskin, 2021).

### Role of Seed Endophytic Bacteria in Seedling Protection Against Fungal Disease

*Bacillus* ssp. (*B. subtilis*, *B. tequilensis*, and *B. velezensis*) and *Pseudomonas aeruginosa* showed strong antifungal activity against fungal phytopathogens including *Fusarium* sp., *Curvularia* sp., *Alternaria* sp., *Rhizoctonia solani*, *Epicoccum sorghinum*, and *Exserohilum rostratum* (Table 3). Fungal pathogens including *Fusarium* spp., *Curvularia* spp., *Alternaria* spp., *Rhizoctonia solani*, and *Exserohilum rostratum* have been reported to decrease crop yield by causing several diseases such as leaf blight, root rot, stalk rot, leaf spot, seedling fall, and grain mold in millet crops (Wilson, 2000; Das, 2017). Several plant-associated bacteria have been reported to show antifungal activity by producing antifungal metabolites, volatile gases, cell wall degrading enzymes, and various types of lipopeptides (Ongena and Jacques, 2008; Gond et al., 2015; Mousa et al., 2016; Jadhav et al., 2017; Sekar et al., 2018; Zhang et al., 2020). SEB KAS2, KAS3, KAS4, KAS5, KAS6, and KAS7 showed protease and KAS5 showed chitinase activity. *Bacillus* spp. are known to have lipopeptide genes and express a variety of antifungal lipopeptides (Ongena and Jacques, 2008). In disc diffusion assays, extracted lipopeptides significantly inhibited the growth of tested fungal phytopathogens (Supplementary Figure 3). Amplification of lipopeptide genes by PCR confirmed the presence of surfactin (Sfp) in *Bacillus subtilis*, surfactin (Sfp) and fengycin (FenD) in *Bacillus tequilensis*, iturin A (ItuD) in *Bacillus velezensis*, and these may be the reason for antifungal activity of the bacteria (Supplementary Table 4). Furthermore, extracted lipopeptides from *Bacillus subtilis* with 2 mg ml^–1^ concentration in PDA, inhibited around 54% of the growth of *Fusarium* sp., and completely checked (MIC) the growth of the fungus at 7 mg ml^–1^ (Figures 5A,B). In a study on maize seed-associated bacteria, Gond et al. (2015) reported that lipopeptides produced by *Bacillus* spp. inhibited *Fusarium moniliforme* growth and stimulated the expression of host defense genes, including PR1 and PR4, which are inhibitory to fungal phytopathogens.

### Mechanism of Antifungal Activity: Microscopic Analysis

Microscopic study revealed that *Bacillus subtilis* and its lipopeptides inhibited the growth of fungal hyphae and spores. Fungi challenged with the bacterium and its lipopeptide showed deformation, swelling, and bulging in hyphae and spores which are clearly visible in bright field (BF), SYTO 9, DAPI, LCB, and calcofluor white (CFW) staining in Figures 6, 7. Live-dead staining with PI alone, and in combination with CFW, indicated that spore and hyphal cells that were swelled/bulged/burst were dead (Figures 6, 7). The swelled hyphal and spore cells further started showing rupturing and leaking out their protoplasm that can be seen in Figure 7. SEM images also confirmed the swelling and bulging/rupturing in spores and hyphae due to lipopeptide treatment (Figure 8). Recently few studies suggested that *Bacillus* lipopeptides inhibit the fungal pathogen by damaging cell walls and cell membranes (Cawoy et al., 2015; Toral et al., 2018). In one study, the lipopeptide surfactin was reported to increase swelling and cytoplasmic leakage and cell death in hyphae of *Magnaporthe grisea* by creating pores in the cell membranes (Wu et al., 2019). Ball-like swellings and vacuolization in hyphae were observed in lipopeptide treated hyphae of *Fusarium moniliforme*, *Botrytis cinerea*, and *Fusarium verticillioides* (Gond et al., 2015; Blacutt et al., 2016; Toral et al., 2018). Lipopeptides create pores in fungal hyphae by depolarizing membranes, inhibiting chitin and glucan synthases, and inducing apoptosis in fungal cells by affecting mitochondrial functions (Kurtz and Douglas, 1997; Qi et al., 2010; Fernández de Ullivarri et al., 2020). Using CFW staining, we observed that burst or swelled portions of the hyphae or spores had blurred images as compared to controls. This may be due to the loss of chitin and glucans from the cell walls (Figure 7). Thus, lipopeptides could be responsible for the damage that results in swelling and pore formation in fungal hyphae and spores. In a microcosm assay for seedling protection, we found that treatment with both *Bacillus subtilis* and its lipopeptide significantly protected seedlings from *Fusarium* infection. Endophytic *Bacillus* spp. have been also reported to induce the expression of defense-related genes of their host plants (Gond et al., 2015; Irizarry and White, 2018).

## Conclusion

The present study shows that pearl millet seeds carry bacterial endophytes which are important for seedling development, establishment, and protection from fungal disease. SEB colonize onto the root surfaces and into root endosphere, mobilize nutrients during germination and growth, and produce antifungal compounds which reduce pathogen infection. This study raises several questions, including: (1) Is there an indirect signaling role of seed endophytes in modulating the expression of developmental and defense genes during seedling development? (2) How do multiple endophytes in seeds interact with each other? (3) What happens in interactions under changing environmental conditions? (4) How may SEB be better utilized in developing microbial products for a sustainable agricultural system?

## Data Availability Statement

The datasets presented in this study can be found in online repositories. The names of the repository/repositories and accession number(s) can be found in the article/Supplementary Material.

## Author Contributions

SV and KK proposed the idea. KK and Anubha performed and designed the experiments. KK and SV wrote the manuscript and prepared the images. GP, AV, and JW contributed to the final editing of the manuscript. All authors contributed to the article and approved the submitted version.

## Conflict of Interest

The authors declare that the research was conducted in the absence of any commercial or financial relationships that could be construed as a potential conflict of interest.

## Publisher’s Note

All claims expressed in this article are solely those of the authors and do not necessarily represent those of their affiliated organizations, or those of the publisher, the editors and the reviewers. Any product that may be evaluated in this article, or claim that may be made by its manufacturer, is not guaranteed or endorsed by the publisher.
