# Supplementary material for: Seed Endophytic Bacteria of Pearl Millet (Pennisetum glaucum L.) Promote Seedling Development and Defend Against a Fungal Phytopathogen
Source: Front Microbiol. 2021 Dec 9;12:774293. doi: 10.3389/fmicb.2021.774293 (PMC8696672; doi:10.3389/fmicb.2021.774293)
Supplement: Supplementary file 1 [file Data_Sheet_1.docx]

**TABLE S1 |** Molecular identification of bacteria re-isolated from root of bacteria treated seedlings with their percentage similarities.

**Bacterial isolates Bacterial isolates from Similarity**

**used for treatment root of treated seedlings Closest matches (%)**

*Kosakonia cowanii* PMR1 *Kosakonia cowanii* 100

*Pantoea stewartii* PMR4 *Pantoea stewartii* 98.27

*Pseudomonas aeruginosa* PMR6 *Pseudomonas aeruginosa* 100

**TABLE S2 |** Enzymatic activities of endophytic bacterial isolates from pearl millet seeds.

Bacterial Amylase Cellulase Pectinase Chitinase Protease

Isolates activity activity activity activity activity

KAS1 ̶ ̶ ̶ ̶ ̶

KAS2 +++ +++ ++ ̶ ++

KAS3 +++ +++ ̶ ̶ +

KAS4 ̶ ̶ ̶ ̶ +

KAS5 ++ ++ ̶ + +

KAS6 ̶ + ̶ ̶ ++

KAS7 +++ +++ ̶ ̶ ++

Where, (-: no activity; + : < 5 mm clear zone; ++: 5 - 10 mm clear zone; +++: more than 10 mm clear zone around endophytic bacterial isolates); KAS1: *Kosakonia cowanii,* KAS2: *Bacillus subtilis,* KAS3: *Bacillus* *tequilensis,* KAS4: *Pantoea stewartii,* KAS5: *Paenibacillus dendritiformis,* KAS6: *Pseudomonas aeroginosa,* KAS7: *Bacillus velezensis*

**TABLE S3 |** List of selected lipopeptide genes and their primer sequences used for PCR amplification and sequencing of lipopeptide genes.

**List of Name of primer and Size of**

**lipopeptide genes their sequences (5’ ̶ 3’) amplicons (bp) References**

Surfactin Sfp-f ATGAAGATTTACGGAATTTA 675 Verma and

(*Sfp*) Sfp-r TTATAAAAGCTCTTCGTACG White, 2018

Bacillomycin Bacc1f GAAGGACACGGAGAGAGTC 875 Verma and

D (*BamC*) Bacc1r CGCTGATGACTGTTCATGCT White, 2018

Iturin A (*ItuD*) ItuD1f GATGCGATCTCCTTGGATGT 647 Verma and

ItuD1r ATCGTCATGTGCTGCTTGAG White, 2018

Fengycin (*FenD*) FenD1f TTTGGCAGCAGGAGAAGTTT 964 Verma and

FenD1r GCTGTCCGTTCTGCTTTTTC White, 2018

**TABLE S4 |** Presences of lipopeptide genes in *Bacillus* ssp. (*Bacillus subtilis, Bacillus tequilensis* and *Bacillus velezensis*) and their closest matches with percentage similarity*.*

**Bcaterial Presence of Closest matches to Similarity (%)**

**Isolates lipopeptide genes**

*Bacillus subtilis* Surfactin (*Sfp*) *Bacillus subtilis* 100

(KAS2)

*Bacillus tequilensis* Surfactin (*Sfp*) *Bacillus* sp. 99.15

(KAS3)

Fengycin (*Fen*D) *Bacillus* sp. 98.56

*Bacillus velezensis* Iturin A (*Itu*D) *Bacillus velezensis* 100

(KAS7)

**FIGURE** **S1 |** Phylogenetic tree of bacterial isolates of pearl millet seeds and their closely related bacterial isolates. The dendrogram was constructed by the MEGA 11 program using the method of Neighbor-Joining based on 16S r DNA gene sequences. Bootstrap values shown at the nodes were based on 1000 replicates. Scale bar represents 0.05 substitutions per nucleotide position.

**FIGURE** **S2 |** All bacterial isolates except KAS5 isolate showed catalase activitiy; control test tube is without bacteria.

**FIGURE** **S3 |** Effects of lipopeptide extracts (From KAS2, KAS3 and KAS7) onto selected fungal phytopathogens through disc diffusion assay. In control, disc contained 20µl methanol only while in the treated group disc were loaded with 20µl of 10µg/µl methanolic solution of lipopeptide (Images have been taken from the plates by stereomicroscope).
