# Supplementary figures and images for: Seed Endophytic Bacteria of Pearl Millet (Pennisetum glaucum L.) Promote Seedling Development and Defend Against a Fungal Phytopathogen
Source: Front Microbiol. 2021 Dec 9;12:774293. doi: 10.3389/fmicb.2021.774293 (PMC8696672; doi:10.3389/fmicb.2021.774293)

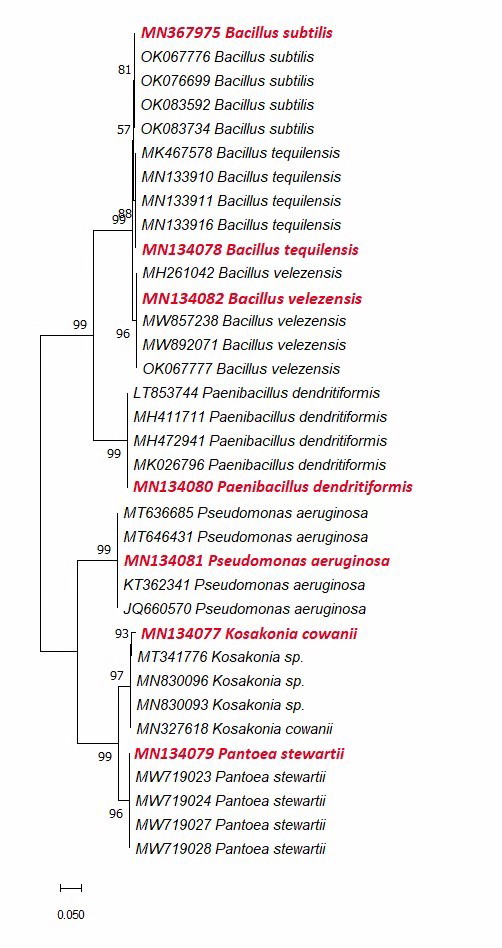

Supplement: Supplementary file 2 [file Image_1.JPEG]

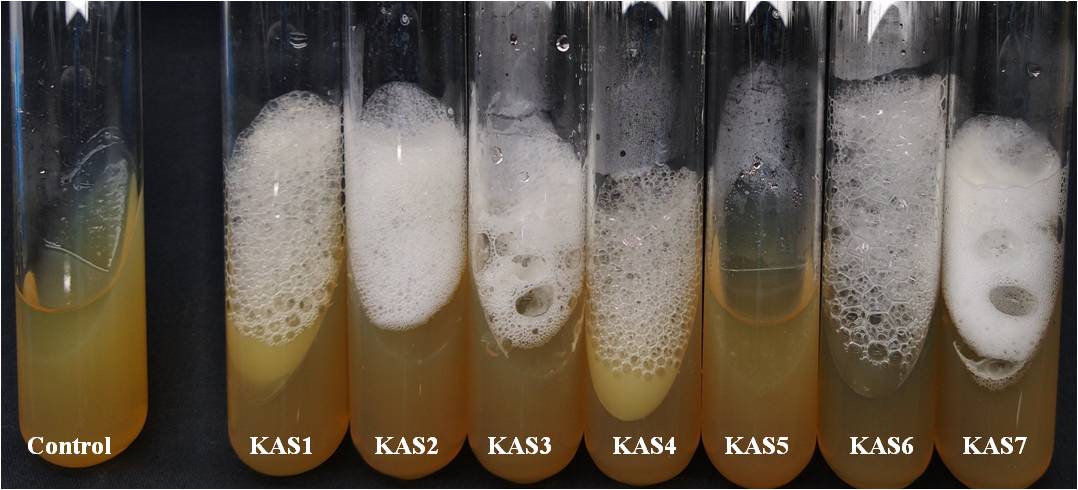

Supplement: Supplementary file 3 [file Image_2.JPEG]

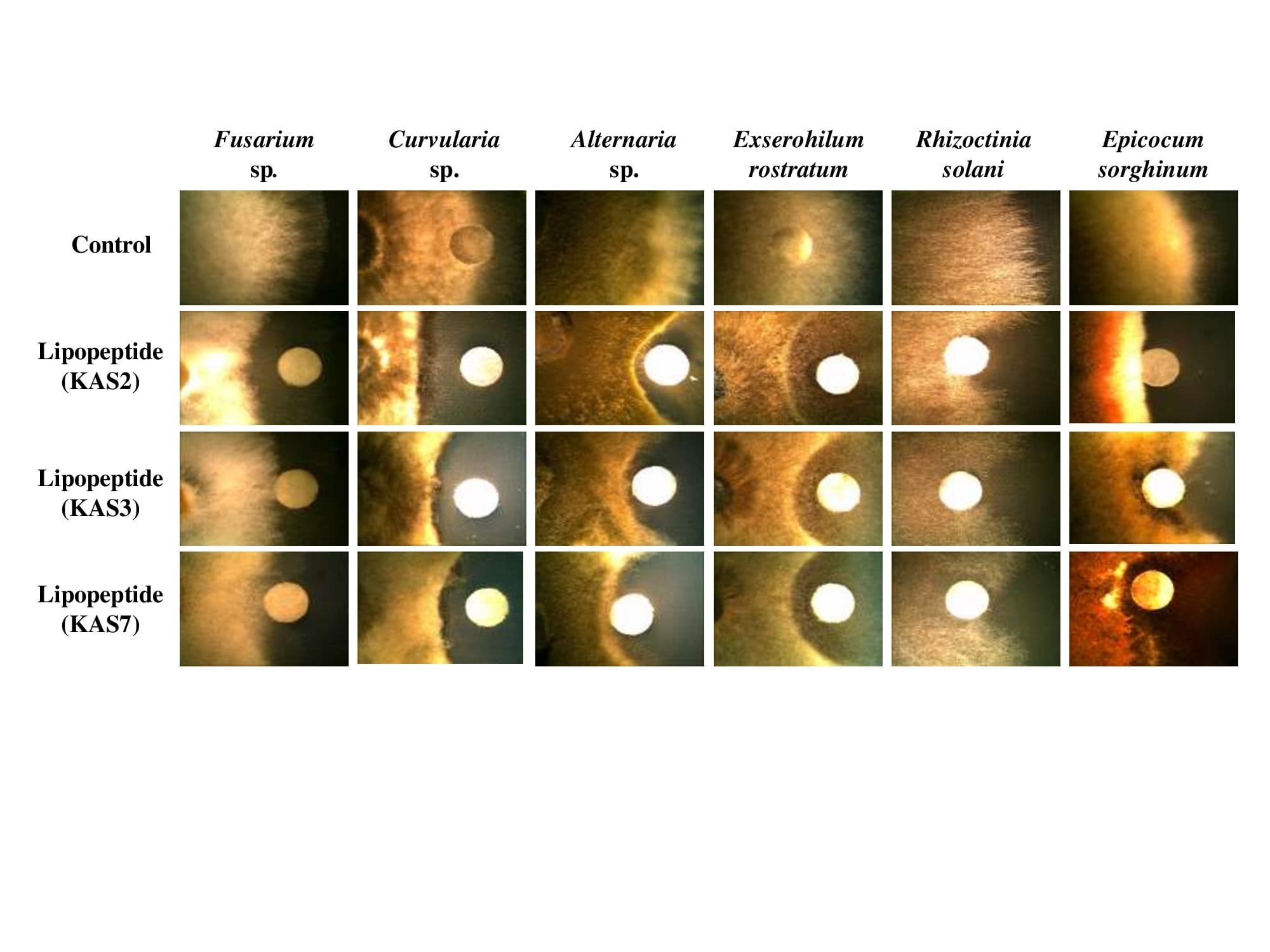

Supplement: Supplementary file 4 [file Image_3.jpg]
